# Supplementary material for: JPA: Joint Metabolic Feature Extraction Increases the Depth of Chemical Coverage for LC-MS-Based Metabolomics and Exposomics
Source: Metabolites. 2022 Feb 26;12(3):212. doi: 10.3390/metabo12030212 (PMC8952385; doi:10.3390/metabo12030212)
Supplement: Supplementary file 1 [file metabolites-12-00212-s001.zip › metabolites-1585395-SM final/JPA_supplementary_JG_20220222.pdf]

Supplementary files

JPA: Joint Metabolic Feature Extraction Increases the Depth of Chemical Coverage for LC-MS-Based Metabolomics and Exposomics

Jian Guo[1], Sam Shen[1], Min Liu[2], Chenjingyi Wang[1], Brian Low[1], Ying Chen[1], Yaxi Hu[1], Shipei Xing[1], Huaxu Yu[1], Yu Gao[3], Mingliang Fang[2], Tao Huan[1]*

[1] Department of Chemistry, Faculty of Science, University of British Columbia, Vancouver Campus, 2036 Main Mall, Vancouver, V6T 1Z1, BC, Canada

[2] School of Civil and Environmental Engineering, Nanyang Technological University, Singapore 639798

[3] Department of Pharmaceutical Sciences, College of Pharmacy, University of Illinois at Chicago, Chicago, IL, 60612, USA

[*] Author to whom correspondence should be addressed:

Dr. Tao Huan

Tel: (+1)-604-822-4891

E-mail: thuan@chem.ubc.ca

Internet: https://huan.chem.ubc.ca/

Table of Contents

Figure. S1. Representative example of bad chromatographic peak shape extracted only by JPA-MS^2 recognition and not JPA-peak picking from the standard mixture.....…………..................S3
Figure S2. Comparison of feature extraction performance of different data processing software …………………………………..…………………………………………… S4
Figure. S3. Tested JPA on urine metabolomics data generated in RP(+) with different cycle times ……………………………………………………………………………… …....S5
Figure S4. Tested JPA using different data processing parameters ………............…………S6
Figure S5. Example EICs of metabolite and exposome standards generated by JPA-PP, JPA-MR, and JPA-TL………………………………………………………………………………S7
Text S1. Detailed dot product algorithm of metabolite annotation in JPA……………………S8
Text S2. Chemicals and solvents…...………………………..………………………………S9
Text S3. The urine sample preparation protocol.……………………………………………S10
Text S4. LC-MS settings for urine metabolomics……………………………………..…S11
Text S5. LC-MS settings for endogenous metabolite standards analysis………………………S12
Text S6. LC-MS settings for exposome standards analysis…………………...……………...S13

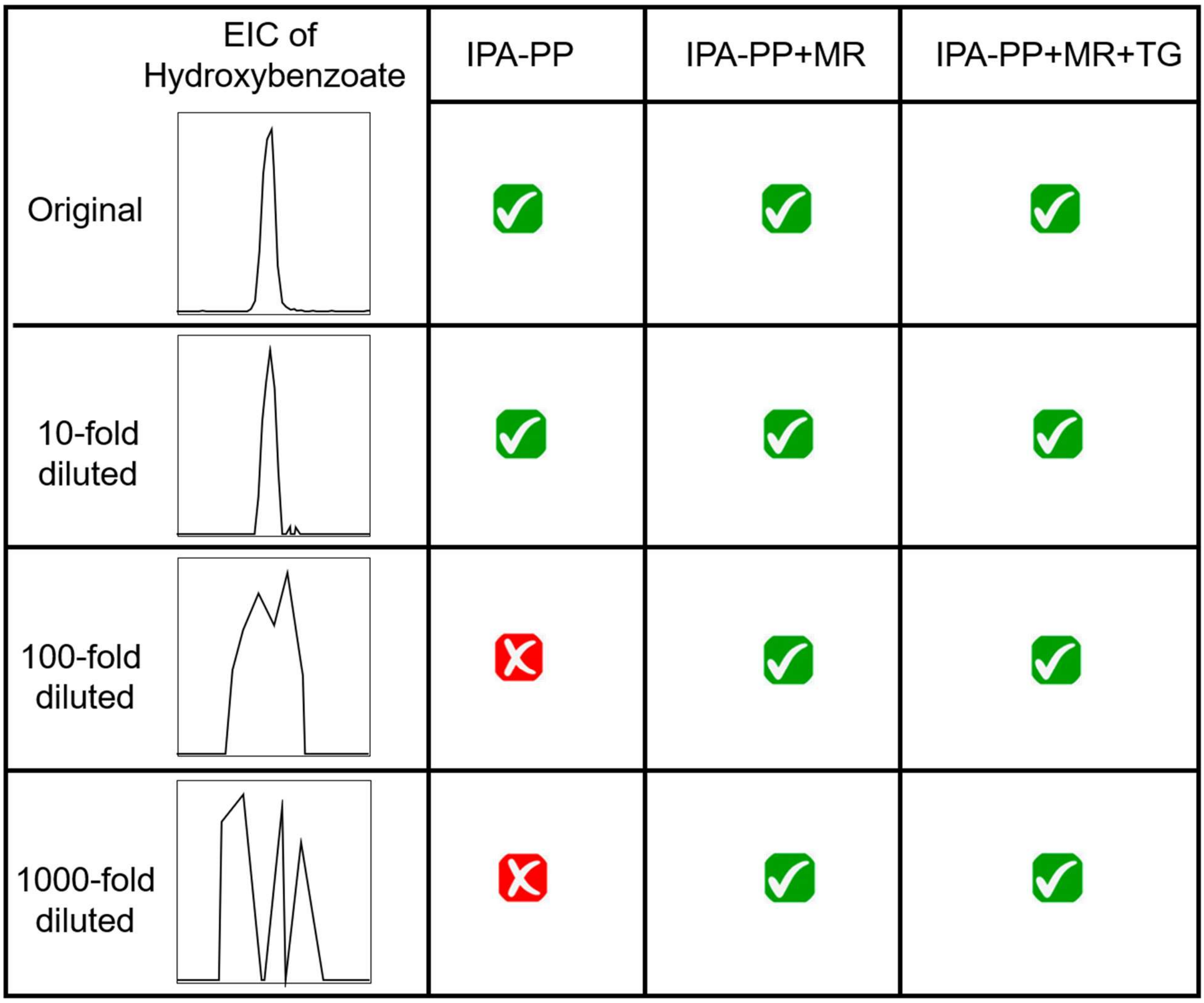

Figure S1. Example of representative bad chromatographic peak shape extracted only by JPA-MS^2 recognition and not JPA-peak picking from the standard mixture.

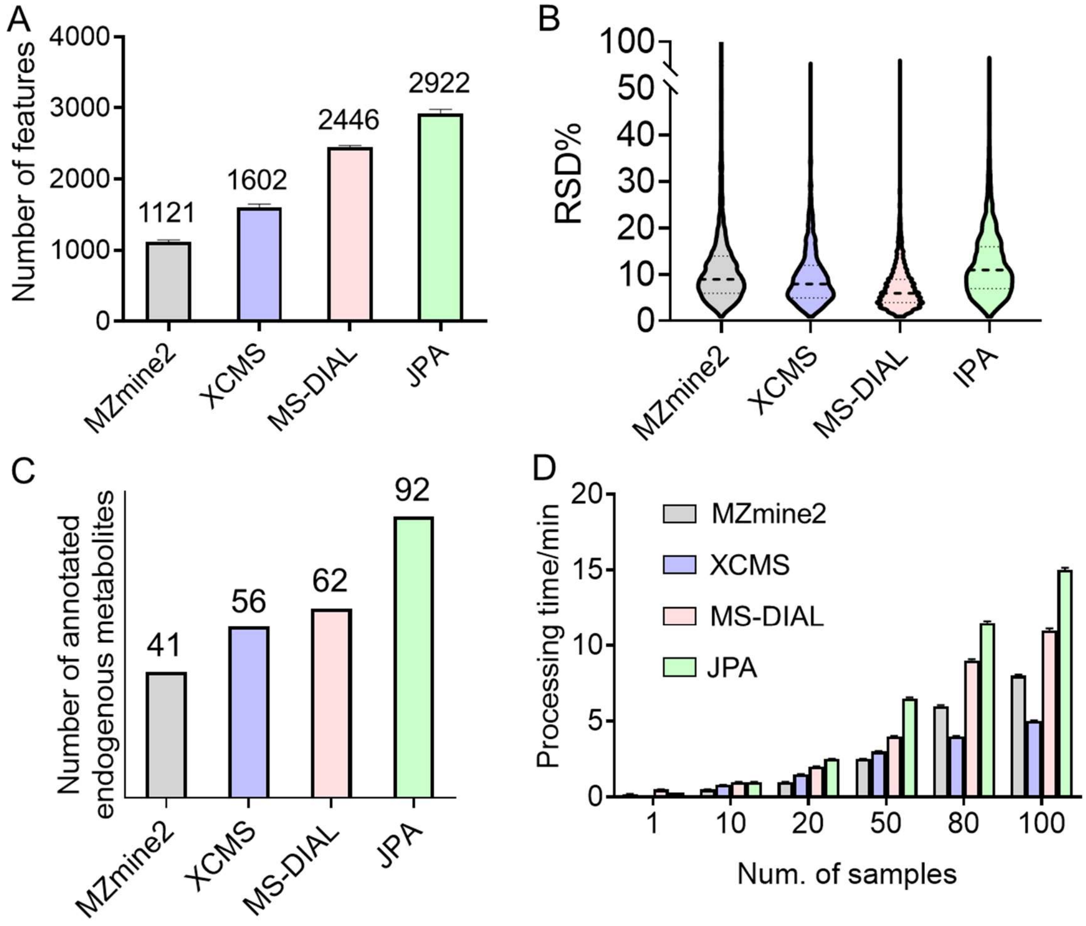

Figure S2. Comparison of feature extraction performance of different data processing software. (**A**) Total number of extracted metabolic features; (**B**) distribution of quantitative RSDs of all metabolic features (RSD was calculated by dividing the standard deviation of the peak height intensities of all features from five urine replicates by the mean); (**C**) the number of annotated endogenous metabolites; (**D**) the processing time of different numbers of samples.

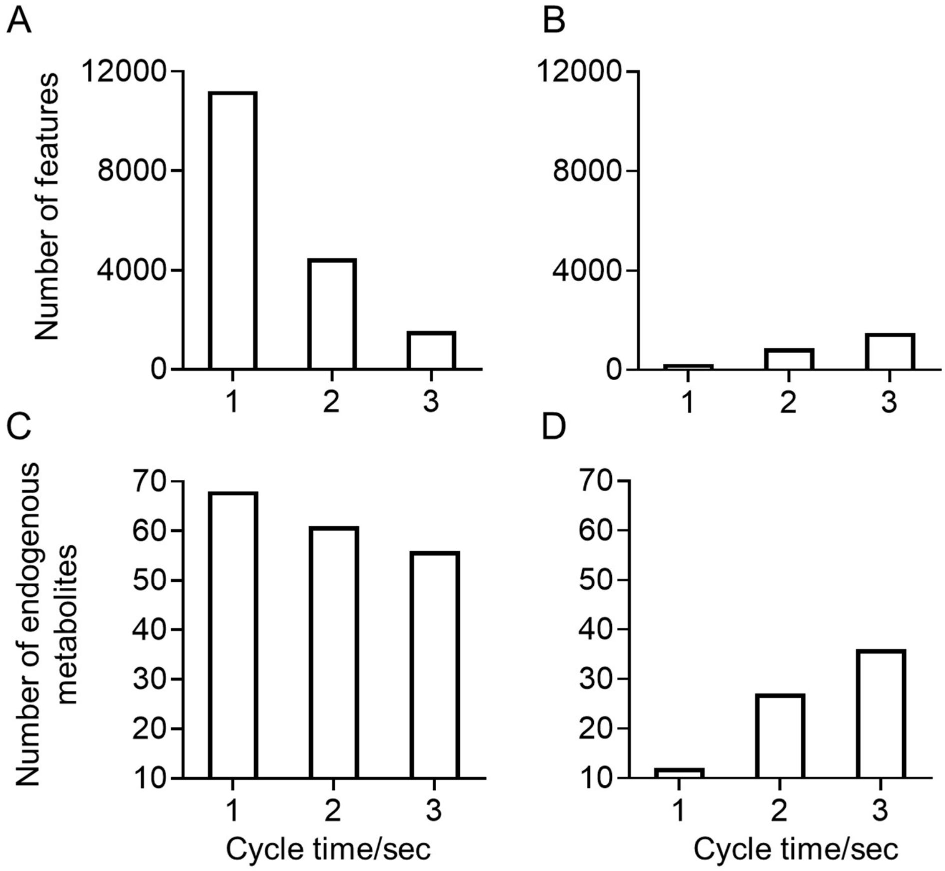

Figure S3. Tested JPA on urine metabolomics data generated in RP(+) with different cycle times. The number of features extracted from (**A**) JPA-PP and (**B**) JPA-MR, and annotated endogenous metabolites from (**C**) JPA-PP and (**D**) JPA-MR.

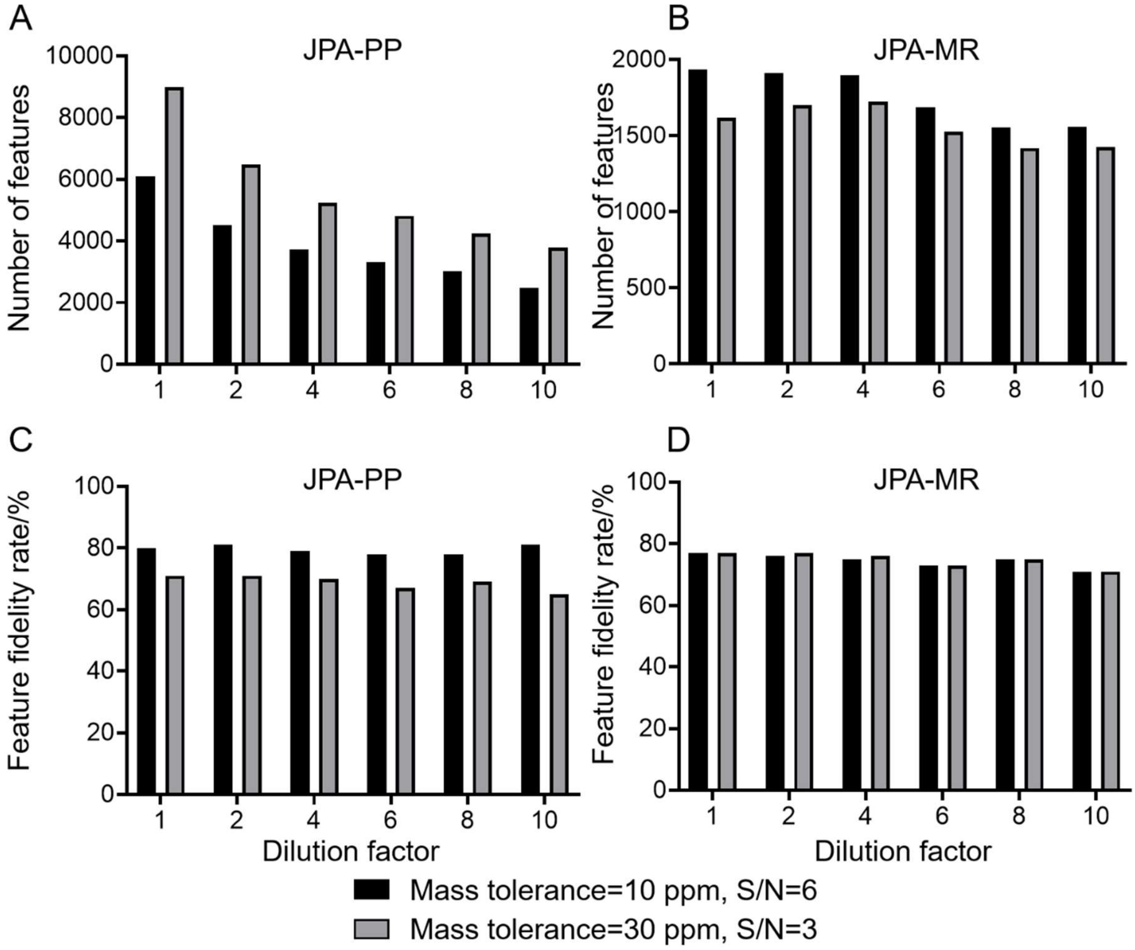

Figure S4. Tested JPA using different data processing parameters. The number of features from (**A**) JPA-PP and (**B**) JPA-MR, and the fidelity rate of features from (**C**) JPA-PP and (**D**) JPA-MR generated by using different data processing parameters (combining RP(+) and HILIC(−) modes).

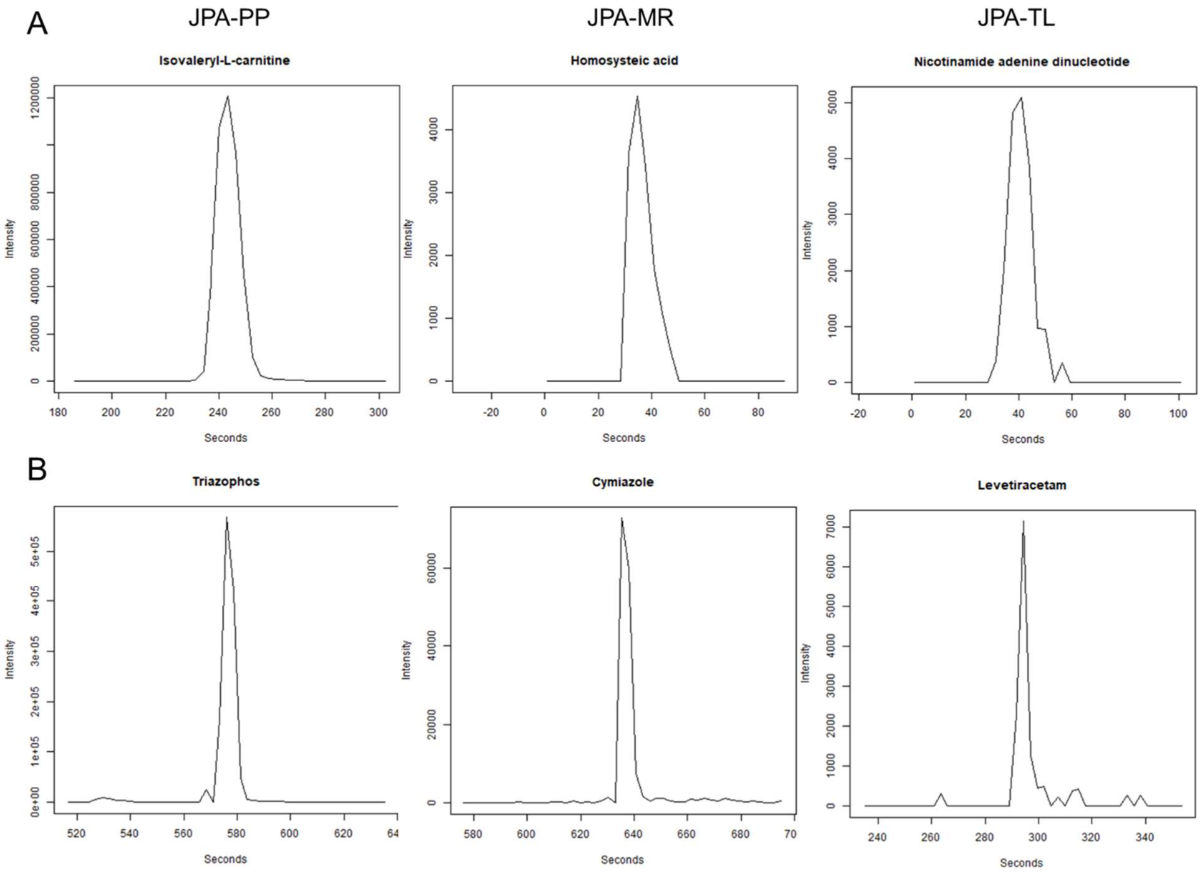

Figure S5. Example EICs of endogenous metabolites (**A**) and exposome standards (**B**) generated by JPA-PP, JPA-MR, and JPA-TL.

Text S1. Detailed dot product algorithm of metabolite annotation in JPA.

Details of dot product calculation:

$$dot\ product = \frac{\sum(W_{act.} \times W_{lib.})}{\sqrt{\sum(W_{act.}{}^{2}) \times \sum(W_{lib.}{}^{2})}}$$

(1)

Dot product calculates the similarity between the experimental MS^2 spectrum and the reference MS^2 spectrum. Before calculating the dot product, the experimental MS^2 spectrum is first matched to the reference MS^2 spectrum based on the fragment *m/z* values to generate a matrix of alignment results. Weighted intensity values (W) are then calculated from the alignment results for all the matched experimental fragment ions and all the reference fragment ions: W_i = (intensity of fragment ion)$_i^n$ × (m/z value)$_i^m$ (n = 1, m = 0 in our case). W_{act} is the vector of weighted intensity values of the experimental MS^2 spectrum that matched to the reference MS^2 spectrum. If a library fragment ion doesn't have matched experimental MS^2 information, the weighted intensity of that fragment ion (W_{act}) will be zero. W_{lib} is the vector of weighted intensity values of all the fragment ions in the library MS^2 spectrum. Dot product is then calculated between W_{act} and $W_{lib,}$ as indicated in equation 1. The dot product ranges from 0 to 1, from no match at all (dot product = 0) to perfect match (dot product = 1).

Text S2. Chemicals and solvents

LC-MS grade water (H_2O), acetonitrile (ACN), methanol (MeOH) and other solvents were purchased from Fisher Scientific. All metabolite standards and other chemicals were purchased from Sigma-Aldrich.

Text S3. The urine sample preparation protocol

The urine sample was collected from a healthy male volunteer. Methanol (MeOH)-based metabolite extraction followed by centrifugation was applied to remove undissolvable matter in the urine sample. In brief, 50 μL of urine was placed in a 1.5 mL Eppendorf vial and mixed with 150 μL of LC-MS grade MeOH. After vortexing, the solution was spun down at 14 000 rpm, and the clear urine supernatant was aliquoted to a clean 1.5 mL Eppendorf vial. The solvent was evaporated using a SpeedVac, and the dried urine sample was reconstituted with 200 μL of acetonitrile/water (ACN/H2O, 1:1, v/v) for LC-MS analysis. The urine sample was then diluted 2, 4, 6, 8, and 10-folds for use.

Text S4. LC-MS settings for urine metabolomics

Five analytical replicates of urine samples were analyzed. The LC-MS analysis was performed on Bruker Impact II™ UHR-QqTOF (Ultra-High Resolution QqTime-Of-Flight) mass spectrometer coupled with the Agilent 1290 Infinity™ II LC system. 2 µL was injected in sequence onto a Waters ACQUITY UPLC BEH C18 Column (130Å, 1.7 µm, 1.0 mm × 100 mm). Mobile phase A was H_2O with 0.1% formic acid, and mobile phase B was ACN with 0.1% formic acid. The chromatographic gradient was run at a flow rate of 0.15 mL/min as follows: 0 min, 95% A; 8 min, 75% A; 14 min, 30% A; 20 min, 5% A; 23 min, 5% A; 23.01 min, 95% A; 30 min, 95% A. The mass spectrometer was operated in Auto MS/MS and positive mode. The ionization source capillary voltage was set to 4.5 kV. The nebulizer gas pressure was set to 1.6 bar. The dry gas temperature was set to 220 °C. The collision energy for MS/MS was set to 20-50 eV. Data acquisition was performed in the range of 40-1200 *m/z* at a frequency of 8 Hz. The charge state was 1. The cycle time was 3 seconds. During MS^2 scanning period, if the precursor ion has an MS signal intensity larger than 1e5, MS^2 spectra rate is 12 Hz. Otherwise, if the precursor ion intensity is below 1e5, MS^2 spectra rate is 4 Hz. The instrument selects the precursors in MS^1 spectra for MS^2 scanning from highest to lowest intensity, until no more precursors are available or the 3 second cycle time is finished. Bruker DataAnalysis was used to calibrate the raw MS spectra and export the raw MS files to .mzXML and .abf data formats for downstream data interpretation.

Text S5. LC-MS settings for endogenous metabolite standards analysis

Three analytical replicates of urine samples were analyzed. The LC-MS analysis was performed on Bruker Impact II™ UHR-QqTOF (Ultra-High Resolution QqTime-Of-Flight) mass spectrometer coupled with the Agilent 1290 Infinity™ II LC system. 2 µL sample solutions of each triplicate were injected in sequence onto a Waters ACQUITY UPLC BEH C18 column (130 Å, 1.7 µm, 1.0 mm × 100 mm) in reversed phase (RP) mode, and a Millipore ZIC-pHILIC column (200 Å, 5 µm particle size, 2.1 × 150 mm) in hydrophilic interaction (HILIC) mode. For internal calibration, 2 µL of 150 mM sodium formate (NaFA) solution was injected. In RP analysis, mobile phase A was H2O with 0.1% formic acid (FA), and mobile phase B was 0.1% FA in ACN. RP gradient was set as follows: 0 min, 95% A; 8 min, 75% A; 14 min, 30% A; 20 min, 5% A; 23 min, 5% A. Post-gradient equilibrium time was 7 min at 95% A. Flow rate was 0.15 mL/min. In HILIC analysis, mobile phase A was 95% H2O and 5% ACN with 10 mM ammonium acetate at 9.8 pH, and mobile phase B was 5% H2O and 95% ACN. HILIC gradient was set as follows: 0 min, 5% A; 20 min, 80% A; 25 min, 95% A. Post-gradient equilibrium time was 19 min at 5% A. The mass spectrometer was operated in Auto MS/MS and positive mode. The ionization source capillary voltage was set to 4.5 kV in positive scanning mode and -3 kV in negative scanning mode. The nebulizer gas pressure was set to 1.6 bar. The dry gas temperature was set to 220 °C. Absolute threshold was set to 250 cts. Collision energy was stepping from 21 to 45 eV. The mass detection range was 50 to 1500 in positive mode and 65 to 1500 in negative mode. The charge state was 1. The cycle time was 3 seconds with the spectra rate of 8 Hz. Bruker DataAnalysis was used to calibrate the raw MS spectra and export the raw MS files to .mzXML and .abf data formats for downstream data interpretation.

Text S6. LC-MS settings for exposome standards analysis

The exposome standard mixture was analyzed on a high-performance liquid chromatography (HPLC) system (1200 series, Agilent Technologies) coupled to a 6550-quadrupole time-of flight (qToF) mass spectrometer (Agilent Technologies, Singapore). The mobile phase A was water with 5 mM ammonium acetate, and mobile phase B was acetonitrile. The linear gradient was set as follows: 0~2.5 min: 5 % B; 2.5~10 min: 5% B to 95% B; 10~12 min: 95% B; 12~15 min: 95% B to 5% B; 15 ~ 17 min: 5% B. Flow rate was 0.5 mL/min, and column temperature was 25 °C. Other parameters were set as follows: gas temperature: 225°C; gas flow: 14 L/min; sheath gas temperature: 350 °C; sheath gas flow: 11 L/min; nozzle voltage: 1000 V; nebulizer pressure: 35 psi; capillary voltage: 3500V for positive (ESI+, electrospray ionization); nozzle voltage: 1000 V; fragmentor: 175 V. The instrument was set to acquire data over the *m/z* range of 50 to 1,000, with the MS acquisition rate of 1 spectra/s. The system was tuned in extended dynamic range mode (2 GHz). Mass calibration was enabled using reference masses of 121.0508 and 922.0098 in ESI (+). For data-dependent acquisition (DDA) auto-MS/MS of selected precursors, the default isolation width was set as narrow (~1.3 *m/z*), with a MS acquisition rate at 6 spectra/s and MS/MS acquisition at 13.33 spectra/s to acquire over the *m/z* range of 50 - 1100. Tandem mass spectrometry (MS/MS) data were acquired at the collision energies of 20 and 40V.
